# Promoting mental health by peer education at the University of Debrecen

**DOI:** 10.1192/j.eurpsy.2024.503

**Published:** 2024-08-27

**Authors:** V. A. Molnár, E. Ménes, K. Kósa, L. I. Tóth

**Affiliations:** ^1^Department of Behavioural Sciences; ^2^Department of Emergency Medicine; ^3^Internal Medicine Building “A”, University of Debrecen, Debrecen, Hungary

## Abstract

**Introduction:**

Medical students have been shown to experience mental health problems more frequently compared to their non-medical student peers. This can manifest in pathological levels of stress and depression, and can lead to substance abuse, reduced academic performance, or even suicide.

**Objectives:**

A credit course on professional socialization is offered for medical students at the University of Debrecen in the form of peer education. Our goal was to evaluate experiences of this course delivered in the past 5 academic years.

**Methods:**

After reviewing the relevant literature, the structure of a focus group interview was developed. The focus group consisted of 8 participants and was moderated by the course supervisor with the help of an assistant moderator. The group summarized the number of students completing the course, and narratives of teaching experiences between 2018/19 and 2022/23. They also revised relevant versions of the tutors’ handbook containing the topics and methodology of the course. The duration of the interview was 90 minutes, and it was tape recorded by the assistant moderator, who also made notes in case the tape is inaudible.

**Results:**

Between 2018/19 and 2022/23 61 students finished the course with the help of 23 tutors. The course is offered for students of general medicine, dentistry and pharmacy to improve their positive professional attitudes and social skills through group work and practical exercises. The medical curriculum includes mandatory courses with practical opportunities for developing professional and social skills, but due to the limited number of contact hours and the varying levels of student interest and motivation, these skills are difficult to master. The credit course was developed using the concept of Bálint groups, offering peer-supervised opportunities for motivated students above year 2 to practice their professional skills in controlled conditions while also receiving feedback from their peer group leaders. The course complements the traditional medical curriculum and sensitizes students in a protected environment in which they can observe their own communication more consciously and recognize unfavourable behaviour patterns. Developing the ability to work in a team, learning to listen, and practicing assertiveness during study years can also reduce performance-related stress and future medical errors along with increasing job satisfaction and patient adherence.

**Conclusions:**

Based on the narrative summary of the focus group, both the experiences of participating students and peer teachers are positive, the handbook is a useful tool. The structured focus group provides a suitable method to evaluate the credit course which should be held once every academic year to evaluate the implemented course and explore options to improve future courses.

**Disclosure of Interest:**

None Declared

